# The association between parental phubbing and pathological digital use among children and adolescents: a meta-analysis of Chinese samples

**DOI:** 10.3389/fpsyg.2026.1883060

**Published:** 2026-09-04

**Authors:** Li Feng, Shuanglong Li

**Affiliations:** 1 College of Education Science, Kashi University, Kashi, China; 2 Regional Basic Education Curriculum and Teaching Innovation Research Team, Kashi University, Kashi, China

**Keywords:** children and adolescents, meta-analysis, moderating effect, parental phubbing, pathological digital use

## Abstract

As a typical manifestation of parental emotional neglect, parental phubbing shows associations with pathological digital use among children and adolescents and is also adversely associated with their physical and mental wellbeing. Nevertheless, existing empirical studies have reached inconsistent conclusions regarding the association between them. Grounded in parental acceptance-rejection theory and compensatory Internet use theory, this study retrieved 50 eligible empirical studies from authoritative databases and adopted a random-effects model to perform a meta-analysis, accompanied by publication bias tests, heterogeneity tests, subgroup analyses, and meta-regression analyses. This study aimed to clarify the magnitude of the correlation between parental phubbing and pathological digital use among children and adolescents and to explore the sources of inconsistent research conclusions and the moderating mechanism of related variables. The results demonstrated a moderate, statistically significant positive association between parental phubbing and pathological digital use among children and adolescents (*r* = 0.308, 95% CI [0.280, 0.336]). The correlation between the two variables was significantly moderated by parental phubbing measures, pathological digital use measures, and behavioral subtypes of pathological digital use. In contrast, no significant moderating effects were detected for only-child status, the proportion of male children and adolescents, age groups, and residential settings [rural versus (vs.) urban].

## 1 Introduction

With the advent of the digital era, digital life has been fully integrated into people’s daily routines (Dai et al., 2024). People’s work, study, entertainment, and other daily activities are highly dependent on digital devices, and the frequent and long-term use of digital devices has become a social norm, which is accompanied by the widespread issue of pathological digital use (Agrawal, 2021). This overarching construct replaces the prevalent but clinically unrecognized label “digital addiction.” Existing empirical studies use subtype measures such as smartphone addiction, Internet addiction, and gaming addiction to measure pathological digital use. Pathological digital use is a kind of addiction-like behavior characterized by compulsive use of mobile phones or the Internet, and it is concurrently associated with adverse physical and mental health outcomes (Dahl and Bergmark, 2020). Specifically, it often co-occurs with interpersonal difficulties such as social isolation, disengagement from offline social activities, and interpersonal conflicts, while also correlating negatively with learning efficiency (Lee et al., 2024). Among various groups affected by pathological digital use, the proportion of children and adolescents has been rising year by year (Xiao et al., 2025). This pattern is observed mainly because children and adolescents are in a critical period of personality shaping and behavior formation. Their self-control ability is not yet fully developed, and they are highly susceptible to imitation and indulgence, which corresponds to higher concurrent levels of pathological digital use in this group in the digital environment (Hu et al., 2025). Relevant statistics show that approximately 13.6% of Chinese children and adolescents spend more than 3 h online every day, among whom 48.9% exhibit mild Internet addiction tendencies (School of Journalism and Communication, Beijing Normal University, 2025). Data released by the World Health Organization Regional Office for Europe (2024) indicate that 11% of children and adolescents across 44 European countries showed problematic social media use, and 12% were at risk of gaming addiction World Health Organization Regional Office for Europe (2024). Adolescent pathological digital use correlates with multiple factors including familial, social, and environmental influences. Among these factors, the family is the most direct and long-term influential context, and parents act as important behavioral role models within the family environment (Atalan Ergin and Essau, 2025). Existing studies have shown that parents spend an average of 0.5 to 7.5 h per day using digital devices, with daily usage reaching as high as 5 h even when their children are present (Blackman, 2015). Furthermore, 73% of parents use digital devices during dinner with their children, and 35% still browse their mobile phones during face-to-face communication with their children (Zhao et al., 2022). Such behaviors are concurrently associated with feelings of neglect in children and adolescents, which correspond to a greater tendency to seek emotional attention and interaction through the Internet, and higher concurrent levels of pathological digital use are reported alongside these experiences (Liu et al., 2024).

However, there is no consistent conclusion regarding the association between parental phubbing and pathological digital use among children and adolescents. Most studies have found a significant positive association between the two (Zhang et al., 2021), while only a small number of studies have failed to detect a significant association (Li et al., 2025). Given the controversial findings in existing research and the lack of integrated studies on the association between parental phubbing and pathological digital use among children and adolescents, this study adopts a meta-analytic approach to explore their association. The aims are to resolve current academic debates, mitigate the bias from individual studies caused by differences in research samples and measurement tools, reveal the overall association strength between the two variables, identify potential moderating factors, and provide empirical evidence for the prevention and intervention of adolescent pathological digital use.

### 1.1 The association between parental phubbing and pathological digital use among children and adolescents

There are two main views on the association between parental phubbing and pathological digital use among children and adolescents:

The first perspective holds that parental phubbing is significantly correlated with pathological digital use among children and adolescents. Through the lens of parental acceptance-rejection theory, parental emotional acceptance or rejection in early childhood closely shapes emotional states and behavioral patterns in childhood and adolescence. In parent–child interactions, excessive parental engagement with digital devices, accompanied by inattention to their children, tends to be perceived by children and adolescents as being neglected, an experience regarded as a form of emotional rejection (Rohner and Lansford, 2017). Perceived emotional neglect is associated with loneliness and low self-worth, which correspond to a greater tendency for children and adolescents to seek emotional comfort via digital products to fulfill unmet affective needs. This psychological pathway may be further elaborated within the compensatory Internet use theory. It is pointed out that when people encounter emotional distress or problems in real life, they tend to resort to the Internet to escape from and alleviate psychological discomfort. When parents exhibit persistent phubbing, children and adolescents fail to obtain sufficient emotional support and psychological satisfaction from the family environment. Lacking effective coping strategies, they are more likely to numb their negative feelings by engaging in excessive Internet use, regarding it as a compensatory coping mechanism, and in turn higher levels of pathological digital use are observed. Meanwhile, parental phubbing tends to weaken parental constraints and guidance on children and adolescents’ use of electronic devices, which corresponds to a lack of clear behavioral boundaries for digital engagement (McDaniel and Radesky, 2018). Within the family context, a lack of emotional fulfillment and inadequate behavioral supervision may increase the likelihood of pathological digital use among children and adolescents.

The second perspective argues that parental phubbing is not significantly correlated with pathological digital use among children and adolescents or that their direct association is extremely weak. On the one hand, as children and adolescents reach a certain age, they spend more time at school than at home and have less interaction with their parents, which corresponds to greater susceptibility to the influence of peer groups or teachers; thus, parental phubbing has no direct association with their pathological digital use (Li et al., 2025). On the other hand, pathological digital use among children and adolescents is driven by the allure of mobile applications, which is the primary cause of their pathological digital use, so the influence of parental phubbing is minimal (Mu et al., 2022). However, there are relatively few studies supporting this perspective. Only a few empirical studies have indicated that the direct association between parental phubbing and pathological digital use among children and adolescents is not significant and that the role of mediating variables is indispensable (Zhou et al., 2025).

In summary, the first perspective that parental phubbing is significantly positively correlated with pathological digital use among children and adolescents is underpinned by multiple theories, with the findings of most empirical studies also validating this position. Therefore, this study proposes Hypothesis 1: Parental phubbing is significantly positively correlated with pathological digital use among children and adolescents.

### 1.2 Moderating variables of the association between parental phubbing and pathological digital use among children and adolescents

Age groups among children and adolescents may moderate the association between parental phubbing and pathological digital use. On the one hand, from the perspective of parental acceptance-rejection theory, children’s interpretation of parental acceptance and rejection cues develops with advancing age. Compared with childhood, adolescence represents a sensitive developmental stage. Adolescents tend to perceive parental phubbing as a form of emotional rejection more intensely (Xie et al., 2019). On the other hand, adolescents in China have greater access to mobile phones and digital devices relative to children (Geng et al., 2021). Unmet emotional demands in real-life parent–child interactions may prompt adolescents to seek emotional compensation through digital devices, a pattern correlated with higher risks of pathological digital use. Taken together, this study proposes Hypothesis 2: Age groups among children and adolescents moderate the association between parental phubbing and pathological digital use among children and adolescents.

Sex differences exist in individuals’ perceptual sensitivity to parental neglect or emotional alienation. Boys are relatively insensitive to changes in the parent–child emotional climate, whereas girls have a stronger need for emotional support during parent–child interactions. Girls are more likely to detect the lack of parental attention associated with parental phubbing and interpret such behavior as emotional snubbing (Lampropoulou and Lianos, 2025). Prolonged exposure to such emotional snubbing may trigger negative emotions among children and adolescents, further motivating them to seek ways to alleviate these negative feelings. Evidence shows that after perceiving alienation in parent–child relationships, girls often rely on digital media to obtain emotional comfort and a sense of companionship; long-term dependence on online channels to relieve negative feelings may increase their vulnerability to pathological digital use (Shao et al., 2024). At the study level, these individual-level gender differences directly translate into variations in effect sizes across different samples. Specifically, since girls are more susceptible to the impact of parental phubbing, samples with a higher proportion of male participants will yield a weaker overall correlation between parental phubbing and pathological digital use. However, scholarly consensus is lacking on whether the proportion of males moderates the association between parental phubbing and pathological digital use among children and adolescents. Accordingly, this study proposes Hypothesis 3: The proportion of males moderates the association between parental phubbing and pathological digital use among children and adolescents.

The proportion of only children may moderate the association between parental phubbing and pathological digital use among children and adolescents. Existing studies have confirmed that non-only children score significantly higher than only children on total scores of pathological digital use (Liu, 2019). Only children receive more care from parents and other family members, and their need for love and belonging could be fully satisfied in real life (Xiao, 2020). Thus, they rarely resort to the Internet to compensate for their deficit needs. Accordingly, this study proposes Hypothesis 4: Only-child proportion moderates the association between parental phubbing and pathological digital use among children and adolescents.

The measurement tools for parental phubbing may exert a moderating effect on the association between parental phubbing and pathological digital use among children and adolescents. Currently, one of the most widely used tools is the Partner Phubbing Scale (PPS), originally developed by Roberts and David for romantic relationships. Designed to assess the negative impacts of phubbing by romantic partners on the quality of daily interactions, the scale yields higher scores for more significant negative impacts and was later directly adapted to measure parental phubbing (Roberts and David, 2016). Considering its applicability to Chinese samples, Ding et al. (2020) translated and revised the scale, replacing the original target group of “partners” with “parents” to tailor it specifically for evaluating parental phubbing, developing the Chinese version of the Parental Phubbing Scale, where higher scores indicate a higher frequency of parental phubbing. As the former is a general-purpose scale and the latter is specifically adapted for parental scenarios, the revision inevitably involved adjustments to the original scale’s dimensional structure (Ding et al., 2020). This may lead to biases in construct validity during practical measurement. Therefore, Hypothesis 5 is proposed: The association between parental phubbing and pathological digital use among children and adolescents is moderated by the measurement tools for parental phubbing.

From the perspective of pathological digital use measurement tools, academia has developed a variety of scales. Among them, the Mobile Phone Addiction Index Scale (MPAI) compiled by Leung (2008) serves as an early general scale. It is assessed across four dimensions including loss of control and withdrawal, with higher scores indicating greater levels of addiction (Leung, 2008). In contrast, scales developed by Hong, Kwon, Xiong, and other scholars focus specifically on adolescent populations. For example, the 32-item Smartphone Addiction Scale (SAS) was developed by Hong et al. (2012); the scale developed by Xiong et al. focuses on the psychological manifestations of addiction and covers four dimensions: withdrawal symptoms, salience behavior, social comfort, and mood change (Xiong et al., 2012). All the scales measure pathological digital use severity based on scores, with higher scores indicating more severe pathological digital use. However, significant differences exist among different scales in terms of the number of items and the definition of core dimensions of pathological digital use. In summary, Hypothesis 6 is proposed: Measurement tools for pathological digital use moderate the association between parental phubbing and pathological digital use among children and adolescents.

## 2 Methods

### 2.1 Literature search and screening

Considering that numerous empirical studies on parental phubbing and pathological digital use among children and adolescents have been published in Chinese journals within the Chinese cultural context, and the research team was proficient in reading both Chinese and English academic literature, this study conducted a literature search across Chinese and English databases.

Since empirical studies have adopted inconsistent measurement constructs falling under the broad scope of pathological digital use, keywords such as smartphone addiction, internet addiction, and problematic gaming were incorporated into the search string to avoid omitting relevant literature. In English databases (Web of Science, Elsevier SD, SpringerLink, and SAGE), the search string was set as (“parental phubbing”) AND (“pathological digital use” OR “mobile phone” OR “smartphone” OR “cell phone” OR digital OR internet). Consistent Boolean logic was applied to Chinese databases (CNKI Journals, CNKI Master’s and Doctoral Dissertations, Wanfang Journals, and Wanfang Dissertations), and the Chinese search string was formulated as parental phubbing AND (pathological digital use OR mobile phone OR digital OR network). Searches across all Chinese and English databases were restricted to the title and abstract fields. Furthermore, to ensure the comprehensiveness of the literature search, manual screening of the reference lists of included studies was conducted as a supplementary search strategy.

**Figure 1:**
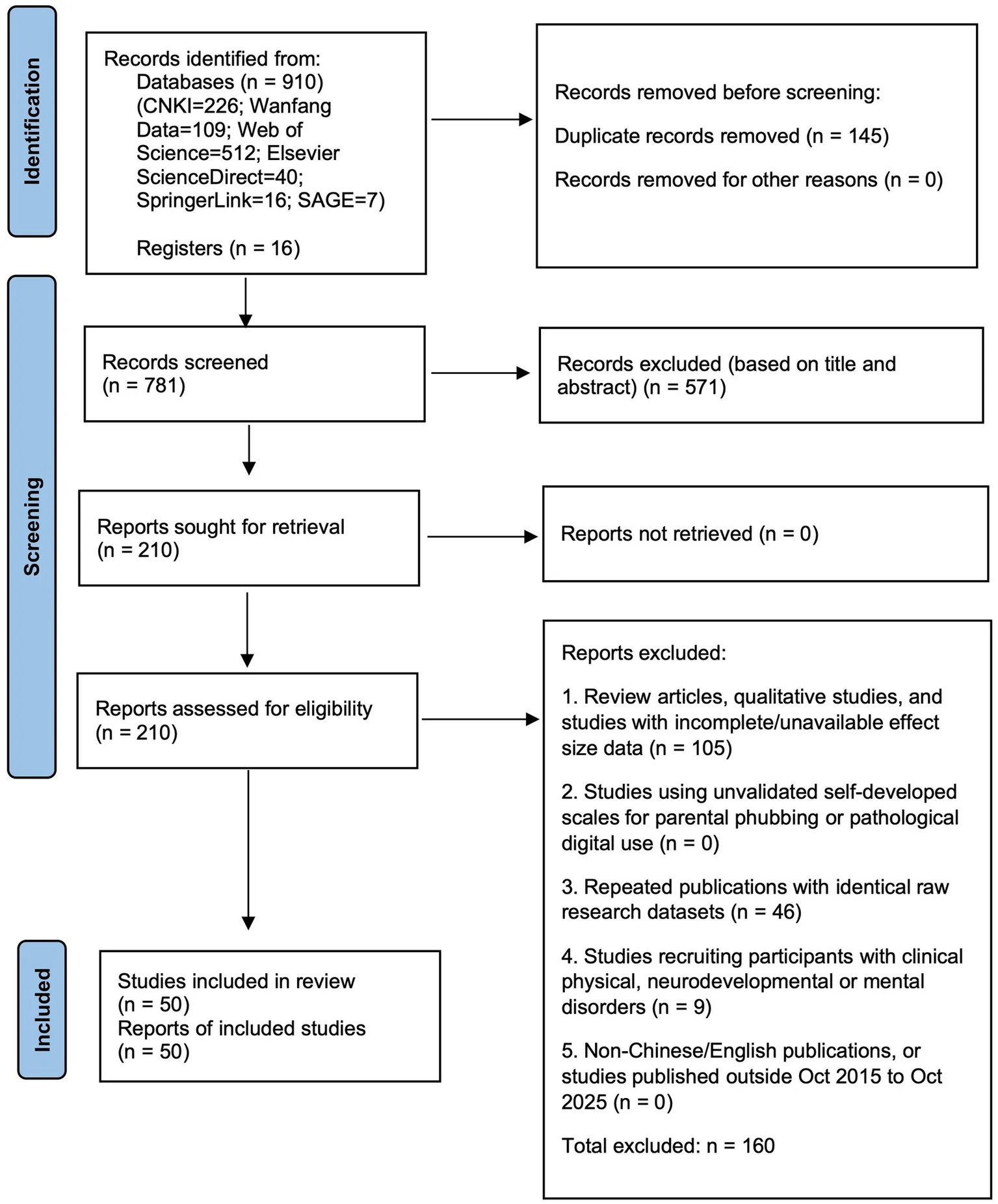
PRISMA flow diagram.

Studies were imported and managed via Zotero, and screening was performed following the PRISMA 2020 statement. The inclusion and exclusion criteria were specified as follows: (1) Studies were quantitative and provided complete statistical data on parental phubbing and pathological digital use among children and adolescents, including clear sample sizes and correlation coefficients r or statistical indicators convertible into r (e.g., standardized regression coefficient *β*), with no apparent data errors. (2) Studies measured both parental phubbing and pathological digital use among children and adolescents using formally validated measurement scales. (3) For multiple publications based on identical datasets, only one complete paper was retained to avoid duplicate extraction of effect sizes. (4) Participants were limited to typically developing children and adolescents aged 6–18 years receiving formal primary and secondary school education. In this study, the term “typically developing” referred to general school student samples without formal psychiatric or clinical screening. Original studies recruited community samples from children and adolescents in primary and secondary schools; mild subclinical emotional symptoms (anxiety, loneliness, and depressive symptoms) and undiagnosed developmental conditions were not grounds for exclusion. However, studies recruiting participants with chronic physical illnesses, neurodevelopmental disorders (autism spectrum disorder, ADHD), or clinical emotional and behavioral disorders (major depression, clinical anxiety, and severe conduct problems) were strictly excluded. Accordingly, only unselected community student samples free of documented clinical diagnoses were included in the present meta-analysis. (5) Manuscripts written in Chinese or English were eligible. The literature search included studies published between 1 October 2015 and 1 October 2025. All searches were completed on 1 November 2025. This 10-year window was selected partially because smartphones underwent rapid technological iteration and gained widespread popularity over the decade. Such shifts might have altered digital device usage habits of parents and youth alike. Two independent coders evaluated the eligibility of all retrieved full-text articles, with only two inconsistent judgments observed. The raw intercoder agreement rate reached 96.15%. Detailed intercoder reliability statistics are presented in Supplementary Table S4. All discrepancies were reconciled through group discussion to reach a consensus, yielding a final sample of 50 independent studies (50 effect sizes, total *N* = 53,304). The full literature screening workflow is illustrated in Figure 1.

### 2.2 Literature coding and effect size conversion

All moderators and hypotheses tested in this meta-analysis were predefined based on relevant theories before literature coding. This study was not preregistered on PROSPERO. During the literature coding and data processing stage, independent effect sizes were regarded as the unit of analysis. Most studies involved only a single effect size, and a few included multiple effect sizes. Two data combination rules were established according to the number of dimensions under the same construct. When a study provided only a single effect size of the core construct, it was used directly. When two subdivided dimensional effect sizes were provided for the same construct, arithmetic means were applied for aggregation. This approach unified multiple correlated effect sizes within one study into a single overall effect size. The specific combination scheme for each included study is summarized in Supplementary Table S1. Standard principles for effect size extraction were defined in this study. Partial correlation coefficients between parental phubbing and pathological digital use among children and adolescents with other variables adjusted were taken as the primary extracted indicators. In the absence of such data, zero-order correlation coefficients were used for analysis. If a study did not report the correlation coefficient between parental phubbing and pathological digital use among children and adolescents but provided the *β* values, these statistics were first converted into correlation coefficients (r) using the corresponding formulas *r* = β × 0.98 + 0.05 (β ≥ 0); *r* = β × 0.98–0.05 (β < 0) before coding, where r is the converted correlation coefficient for meta-analytic synthesis, and β is the standardized regression coefficient reported in the primary study (Card, 2012). Overall, zero-order Pearson’s correlation coefficients accounted for most extracted effect sizes, and only a small number of primary studies reported partial correlations adjusted for confounding variables.

All eligible studies were coded in this study, including title, first author and publication year, geographic region of the sample, sample size, Pearson’s correlation coefficient between parental phubbing and pathological digital use among children and adolescents, parental phubbing measurement tools, and pathological digital use measurement tools (see Table 1).

**Table 1:** Literature coding.

Author and date	N	SSG	Male Prop	OCP	r(k)	PPM	DAM	Aggregation method
Pan (2025)	970	PreS	50.93%	——	0.382, 1	PPS, Ding et al. (2020)	SSAA, 2018	Single
Deng (2025)	805	JunS	52.80%	46.58%	0.340, 1	PPS, Ding et al. (2020)	OGAS, Yu et al. (2015)	Single
Zhou et al. (2025)	1,230	JunS; SenS	45.77%	60.57%	0.130, 1	PPS, Ding et al. (2020)	MGA, Li	Single
Liang et al. (2025)	1925	SenS	46%	5.2%	0.320, 1	PPS, Ding et al. (2020)	SAS, Kwon	Single
Yang et al. (2025)	5,785	SenS	48.78%	80.86%	0.210, 1	PPBQ, Wang (2020)	SFVAS, Choi et al. (2016)	Single
Liu et al. (2025)	591	JunS; SenS	57.53%	-	0.230, 1	PPS, Ding et al. (2020)	MPAS, Hong et al. (2012)	Single
Gu et al. (2025)	757	SenS	47.80%	-	0.600, 1	PPS, Ding et al. (2020)	MPAS, Hong et al. (2012)	Single
Wang et al. (2025)	1,115	JunS	47.50%	22.3%	0.280, 1	PPS, Roberts and David (2016)	SNAIS, Li et al. (2016)	Single
Xing et al. (2024)	944	JunS	44.90%	-	0.280, 1	PPS, Ding et al. (2018)	MPA, Leung (2008)	Single
Sui and Liu (2024)	502	SenS	33.86%	-	0.442, 1	PPS, Ding et al. (2020)	SASV, Tao	Single
Xu (2024)	1,138	PreS; JunS; SenS	46.90%	-	0.390, 1	PPS, Ding et al. (2020)	SFVAS, Zhang	Single
Jin et al. (2024)	808	JunS	49.09%	18.36%	0.230, 1	PPS, Ding et al. (2020)	OGAS, Ding	Single
Dai et al. (2024)	1912	PreS; JunS; SenS	52.50%	-	0.226, 1	PPS, Ding et al. (2020)	IASC, Young (1998)	Arithmetic mean
Tang et al. (2024)	742	JunS; SenS	54.45%	-	0.420, 1	PPS, Liu (2019)	MPAS, Wang et al. (2020a)	Single
Ma et al. (2024)	786	JunS; SenS	53.10%	-	0.520, 1	PPS, Xie and Xie (2020)	MPAI, Leung (2008)	Single
Li et al. (2025)	315	SenS	57%	-	0.118, 1	PPS, Junttila and Vauras (2009)	IGAS, Skinner et al. (2005)	Single
Zhang et al. (2024)	871	JunS	54.76%	-	0.480, 1	GSBP, Chot	GSP	Single
Zhang (2023)	1,022	PreS	43.35%	——	0.370, 1	PPS, Roberts and David (2016)	mpus, Hong et al. (2019)	Single
Liu (2023)	820	PreS	53.41%	——	0.295, 1	PPS, Ding et al. (2020)	SABAS, csibi, 2016	Arithmetic mean
Qiu (2023)	728	JunS	49.73%	47.12%	0.320, 1	PPS, Ding et al. (2020)	SAS, Kwon	Single
Ma (2023)	1905	SenS	64.70%	——	0.420, 1	PPS, Roberts and David (2016)	MPA, Leung (2008)	Single
Zhu (2023)	430	JunS	46.67%	——	0.310, 1	PPS, Ding et al. (2018)	MAPI, Liang	Single
Yuan (2023)	444	JunS	50%	15.5%	0.404, 1	PPS, Ding et al. (2020)	GS-PB, 2018	Arithmetic mean
Xue (2023)	1,222	PreS	49.30%	17.9%	0.280, 1	PPS, Ding et al. (2020)	MPAS, Hong et al. (2012)	Single
Lü et al. (2023)	2,233	JunS	49.04%	-	0.281, 1	PPS, Ding et al. (2020)	MPAI, Huang	Single
Deng and Hong (2023)	855	JunS	53.33%	-	0.136, 1	PPS, Ding et al. (2020)	IASC, Young (1998)	Single
Tian (2023)	699	JunS	50.80%	18.5%	0.270, 1	PPS, Ding et al. (2020)	IGAS, Ma	Single
Mi et al. (2023)	780	JunS; SenS	50.38%	-	0.540, 1	PPS, Ding et al. (2020)	SAS, Xiong et al. (2012)	Single
Chen et al. (2023)	728	JunS; SenS	49.73%	-	0.320, 1	PPS, Roberts and David (2016)	SAS, Xiang et al. (2019)	Single
Dong et al. (2023)	2,348	JunS	49.40%	-	0.250, 1	PPS, Wang et al. (2020)	FIQ, Wang et al. (2018)	Single
Wang et al. (2023)	2,260	JunS	49.65%	-	0.245, 1	PPS, Wang et al. (2020)	SASSV, Wang et al. (2019)	Arithmetic mean
Yang (2022)	944	JunS	44.92%	12.5%	0.280, 1	PPS, Ding et al. (2020)	MPA, Leung (2008)	Arithmetic mean
Wu (2022)	780	JunS	50.40%	60.8%	0.412, 1	PPS, Ding et al. (2018)	SAS, Xiong et al. (2012)	Single
Ding Q. et al. (2022) and Ding Y. et al. (2022)	812	JunS	42.73%	-	0.320, 1	PPS, Ding et al. (2020)	MPAS, Hong et al. (2012)	Single
Yang et al. (2022)	333	JunS	52.3%	14.4%	0.437, 1	PPS, Ding et al. (2020)	MPAS, Hong et al. (2012)	Single
Ji H. Y. (2022)	799	PreS	58.30%	-	0.370, 1	PPS, Ding et al. (2018)	APQ, Zhang and Zhang (2020)	Single
Ji X. Y. (2022)	2090	SenS	44.40%	-	0.240, 1	PPS, Ding et al. (2018)	SAS, Kwon	Single
Zhou et al. (2022)	1,021	PreS; JunS; SenS	55.70%	-	0.180, 1	PPS, Roberts and David (2016)	POGU, Gentile (2009)	Single
Wang and Lei (2022)	549	SenS	55%	-	0.270, 1	PPS, Ding et al. (2020)	IASC, Young (1998)	Single
Mu et al. (2022)	242	JunS; SenS	34%	-	0.107, 1	PPS, Ding et al. (2018)	SNS, Chen (2019)	Single
Liu (2021)	822	JunS	50.10%	27.2%	0.255, 1	PPS, Ding et al. (2018)	MPAS, Hong et al. (2012)	Arithmetic mean
Geng et al. (2021)	1,447	PreS; JunS; SenS	39.50%	17.9%	0.220, 1	PPS, Ding et al. (2020)	SAS, Kwon	Arithmetic mean
Zhang et al. (2021)	471	JunS	40.13%	-	0.230, 1	PPS, Roberts and David (2016)	MPAS, Hong et al. (2012)	Single
Xiao (2020)	452	SenS	42.50%	54.6%	0.250, 1	PPS, Xiao (2020)	SAS, Xiong et al. (2012)	Single
Ding et al. (2020)	471	JunS	40.13%	-	0.232, 1	PPS, Ding et al. (2020)	MPAS, Hong et al. (2012)	Single
Fu et al. (2020)	2,238	JunS	49.60%	-	0.300, 1	PPS, Roberts and David (2016)	MPDS, Seo et al. (2016)	Single
Niu et al. (2020)	726	JunS; SenS	51.45%	-	0.270, 1	PPS, Roberts and David (2016)	MPAI, Leung (2008)	Single
Ding et al. (2019)	555	JunS; SenS	52.43%	-	0.240, 1	PPS, Roberts and David (2016)	MPAS, Hong et al. (2012)	Single
Liu et al. (2019)	605	SenS	48.24%	-	0.300, 1	PPS, Roberts and David (2016)	Mpd, Dong et al. (2009)	Single
Ding et al. (2018)	339	JunS	54.87%		0.210, 1	PPS, Roberts and David (2016)	MPAS, Hong et al. (2012)	Single

Author and Date, first author and publication date; Nationality, participant nationality; N, sample size; SSG, school stage grouping; Male Prop, male student proportion; OCP, only-child proportion; r, correlation coefficient; Single, single effect size; PPM, parental phubbing measurement tool; DAM, pathological digital use measurement tool; PreS, primary school; JunS, junior high school students; PPS, Parental Phubbing Scale; MPAI, Mobile Phone Addiction Index; SSAA, Scale of Smartphone Application Addiction; SAS, Smartphone Addiction Scale; MPA, Mobile Phone Addiction Index Scale; GS-PB, General Scale of Phubbing Behavior; SASV, Smartphone Addiction Scale-Short Version; MGA, Mobile Game Addiction Scale; PPBQ, Parental Phubbing Behavior Questionnaire; DIS, dependency intention scale; FIQ, Facebook Intrusion Questionnaire; IGAS, Internet gaming addiction Scale; SASSV, Smartphone Addiction Scale-Short Version; GSP, Generic Scale of Phubbing; Mpd, mobile phone dependency; SFVAS, Short-Form Video Addiction Scale; MPATS, Mobile Phone Addiction Tendency Scale; ASD, adolescent smartphone dependence; IAS, Internet Addiction Scale; IGDS9SF, Internet Gaming Disorder Scale—Short Form; POGU, Problematic Online Game Use scale; MPAS, Mobile Phone Addiction Scale; GSBP, Generic Scale of Being Phubbed; SABAS, Smartphone Application-Based Addiction Scale; OGAS, Online Game Addiction Scale; APQ, Adolescent Phubbing Questionnaire; MGAS, Mobile Game Addiction Scale; EUSVS, excessive use of short video scales.

### 2.3 Publication bias test and control

In this study, published journal articles and unpublished master’s and doctoral theses were included. In addition, the methodological quality of included studies was assessed using the JBI critical appraisal checklist, with detailed results presented in Supplementary Table S3. A stepwise analysis strategy of testing, validation, and correction was adopted to examine publication bias. First, a funnel plot was used to judge the presence of publication bias based on the dispersion and symmetry of effect sizes. Second, Egger’s regression test was conducted to further statistically verify the existence of publication bias. Third, the fail-safe N was used for quantitative assessment, which calculates the number of unpublished studies required to turn the pooled effect size from significant to non-significant, so as to evaluate the robustness of the meta-analysis results. Finally, to address potential bias indicated by the funnel plot, the trim-and-fill method was applied to estimate and impute missing effect sizes, and pooled effect sizes were recalculated after correction to improve the accuracy and credibility of the meta-analysis results.

### 2.4 Model selection

In meta-analytic research, two conventional models are available for pooled effect size calculation: the fixed-effects model and the random-effects model. The fixed-effects model assumes that the true effect size is constant across studies and that variation originates solely from sampling error, which is typically applied under high homogeneity conditions. The random-effects model presumes that included studies are randomly sampled from the overall population and that true effect sizes allow for cross-study random variation, which is generally adopted when substantial heterogeneity exists.

Based on previous empirical evidence, the sex ratio and age groups of children and adolescents, only-child proportion, and measurement instruments may moderate the association between parental phubbing and pathological digital use among children and adolescents. Heterogeneity testing was further performed to determine the appropriate analytic model. The magnitude of heterogeneity was evaluated using the Q statistic and I^2^ index. The I^2^ value represents the proportion of between-study variance in total variance. Generally, I^2^ > 75% indicates high heterogeneity, and a significant Q statistic (*p* ≤ 0.05) suggests substantial cross-study heterogeneity. Under such conditions, the random-effects model was adopted for pooled estimation; otherwise, the fixed-effects model was utilized.

### 2.5 Data processing

The effect size index used in this study is the Pearson correlation coefficient r; the software employed for data analysis is Comprehensive Meta-Analysis V3.3. Two approaches were adopted for the analysis of moderating variables: If a moderating variable was a categorical variable, subgroup analysis was conducted to examine the significance of the results; if it was a continuous variable, meta-regression analysis was used to assess the significance of the results. In accordance with the recommendations of existing studies, the number of effect sizes in each subgroup was required to be no fewer than 3 (Song et al., 2014).

## 3 Results

### 3.1 Publication bias test

**Figure 2:**
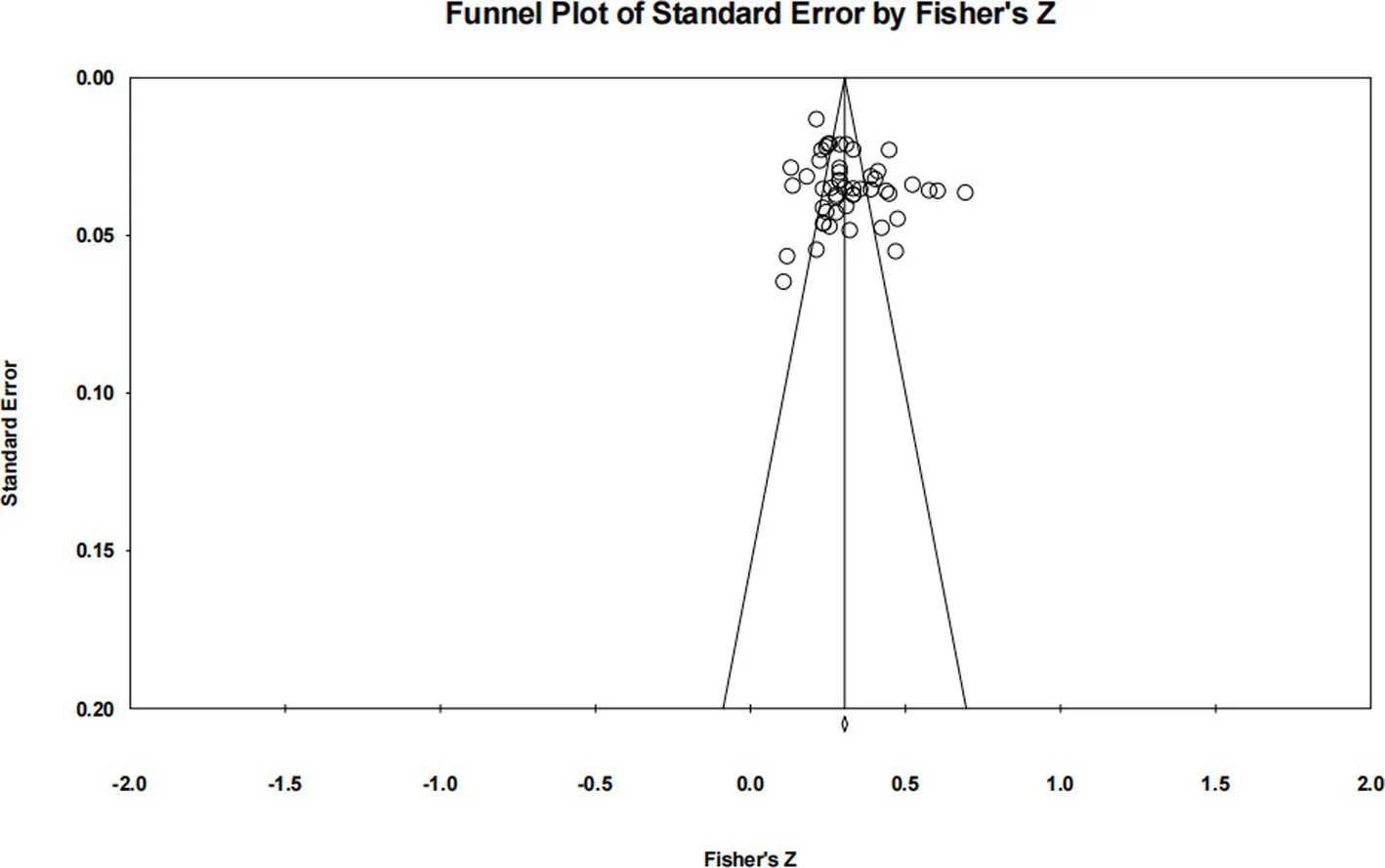
Funnel plot.

**Figure 3:**
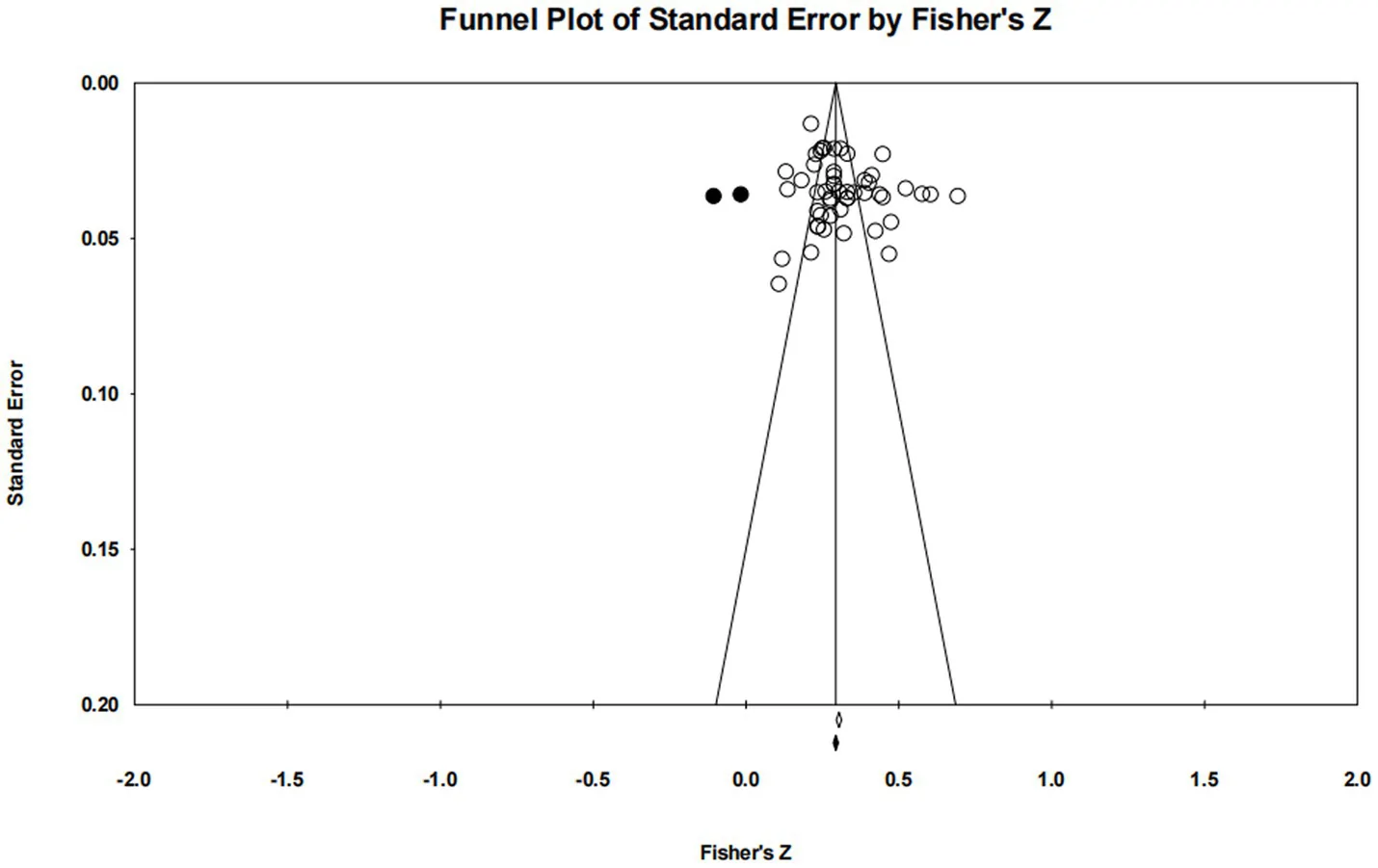
Trim-and-fill funnel plot based on Fisher’s *Z* transformation.

Multiple complementary methods were used to assess publication bias. First, visual inspection of the funnel plot revealed a roughly symmetric distribution of effect sizes, providing preliminary, suggestive evidence against severe publication bias (see Figure 2). To complement the subjective visual judgment of the funnel plot, Egger’s regression test was conducted. The regression intercept was 2.879, with a two-tailed *p*-value of 0.065, marginally above the conventional 0.05 threshold, indicating no strong statistical evidence of substantial publication bias. The trim-and-fill method was then adopted to evaluate potential bias arising from missing studies. The analysis identified two missing studies located on the left side of the mean effect size (see Figure 3). Under the random-effects model, the original pooled correlation coefficient was 0.308 (95% CI [0.280, 0.336]); after correction, the adjusted pooled effect size decreased slightly to 0.295 (95% CI [0.263, 0.326]). The confidence interval still excluded zero, indicating that potential publication bias exerted limited influence on the core findings. Finally, Rosenthal’s classic fail-safe N was calculated as 10,873, which well exceeded the critical value of 5 k + 10 (260). This indicated that a large number of additional non-significant unpublished studies would be required to alter the overall statistical outcome, lending support to the relative stability of the meta-analytic conclusions. Overall, although the trim-and-fill analysis suggested the existence of a small number of missing studies, the magnitude and statistical significance of the pooled effect remained essentially unchanged after correction. These findings indicated that any potential publication bias would not fundamentally distort the core findings of this meta-analysis.

### 3.2 Heterogeneity test

Heterogeneity tests of effect sizes were performed to select suitable analytical models and confirm the need for subgroup analyses and meta-regression analyses. The results revealed Q = 627.466, df = 49, *p* < 0.001, and I^2^ = 92.191%. The I^2^ value exceeded the commonly used cutoff of 75%, indicating high heterogeneity across included studies (Higgins et al., 2003). The 95% prediction interval was markedly wider than the confidence interval, showing substantial dispersion of effect sizes, and relevant outcomes are presented in Table 2. Given the high overall heterogeneity, the random-effects model was adopted for subsequent analyses. Subgroup analyses and meta-regression were further conducted to systematically explore the sources of high between-study heterogeneity.

**Table 2:** Heterogeneity test and pooled correlation estimates (random-effects model).

Outcome variable	Heterogeneity				95%CI		95% PI		Tau-squared	
	Q	*df*	*p-*value	*I* ^2^	Lower	Upper	Lower	Upper	Tаu-squared	Standard error
Correlation	627.466	49	0.000	92.191	0.280	0.336	0.097	0.519	0.011	0.003

Correlation, correlation between parental phubbing and adolescent pathological digital use. All analyses were based on the random-effects model. Q, Cochran’s Q statistic for heterogeneity; df, degrees of freedom; I^2^, proportion of total variance attributable to between-study heterogeneity; 95% CI, 95% confidence interval of the pooled correlation coefficient; 95% PI, 95% prediction interval for the true effect size of a single future study; Tau-squared, estimated between-study variance component.

### 3.3 Main effect test and sensitivity analysis

**Figure 4:**
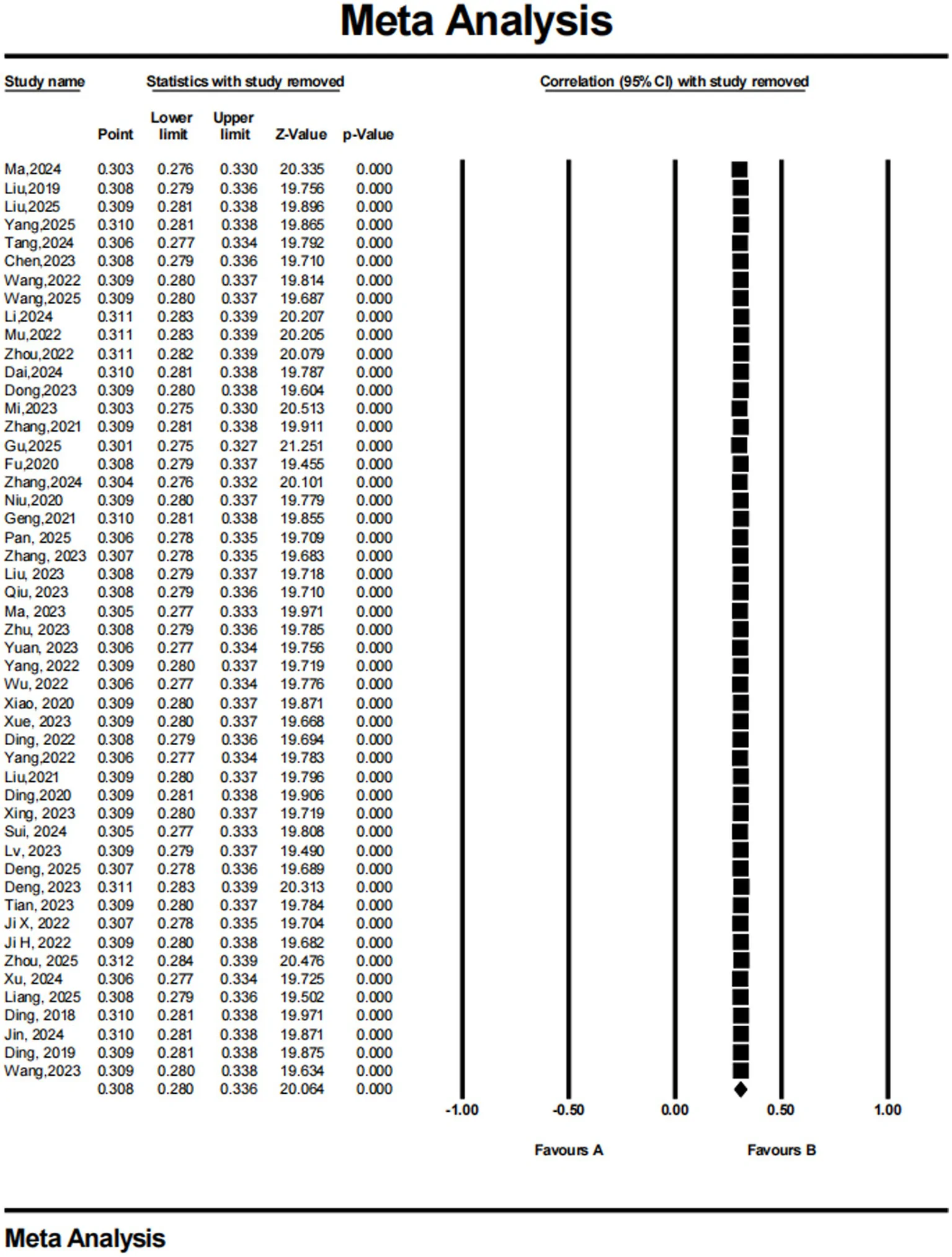
Sensitivity analysis of the main effect between parental phubbing and pathological digital use among children and adolescents.

A total of 50 effect sizes were analyzed under the random-effects model to estimate the association between parental phubbing and pathological digital use among children and adolescents. The pooled correlation coefficient was 0.308, with a 95% confidence interval of [0.280, 0.336]. Classification criteria for correlation coefficients are as follows: *r* = 0.1 indicates a weak correlation, *r* = 0.3 a moderate correlation, and *r* = 0.5 a strong correlation (Muller, 1989). A statistically significant moderate positive association was observed between parental phubbing and pathological digital use among children and adolescents, which verified Hypothesis 1. Sensitivity analysis was performed by omitting one single study at a time to examine result robustness. The pooled effect sizes fluctuated from 0.301 to 0.312. All 95% confidence intervals excluded zero, and all *p*-values were less than 0.001. Effect sizes and their corresponding confidence intervals were tightly distributed, and no obvious outliers were observed (see Figure 4).

### 3.4 Moderating effect test

Subgroup analysis and meta-regression were used to explore factors influencing the association between parental phubbing and pathological digital use among children and adolescents. Analyses of moderating variables revealed the following findings:

The age groups of children and adolescents did not significantly moderate the association between parental phubbing and pathological digital use among children and adolescents. Age groups were treated as a categorical moderator, with participants categorized into primary school, junior high school, and senior high school groups for subgroup analysis. Studies featuring mixed-age samples that were unclassifiable into one specific age bracket were excluded from the subgroup analysis, yielding a final set of 35 independent effect sizes. The between-group heterogeneity test was non-significant (Q = 1.337, df = 2, and *p* = 0.513), indicating no statistically significant differences in effect sizes across age groups. Hypothesis 2 was not supported.

**Figure 5:**
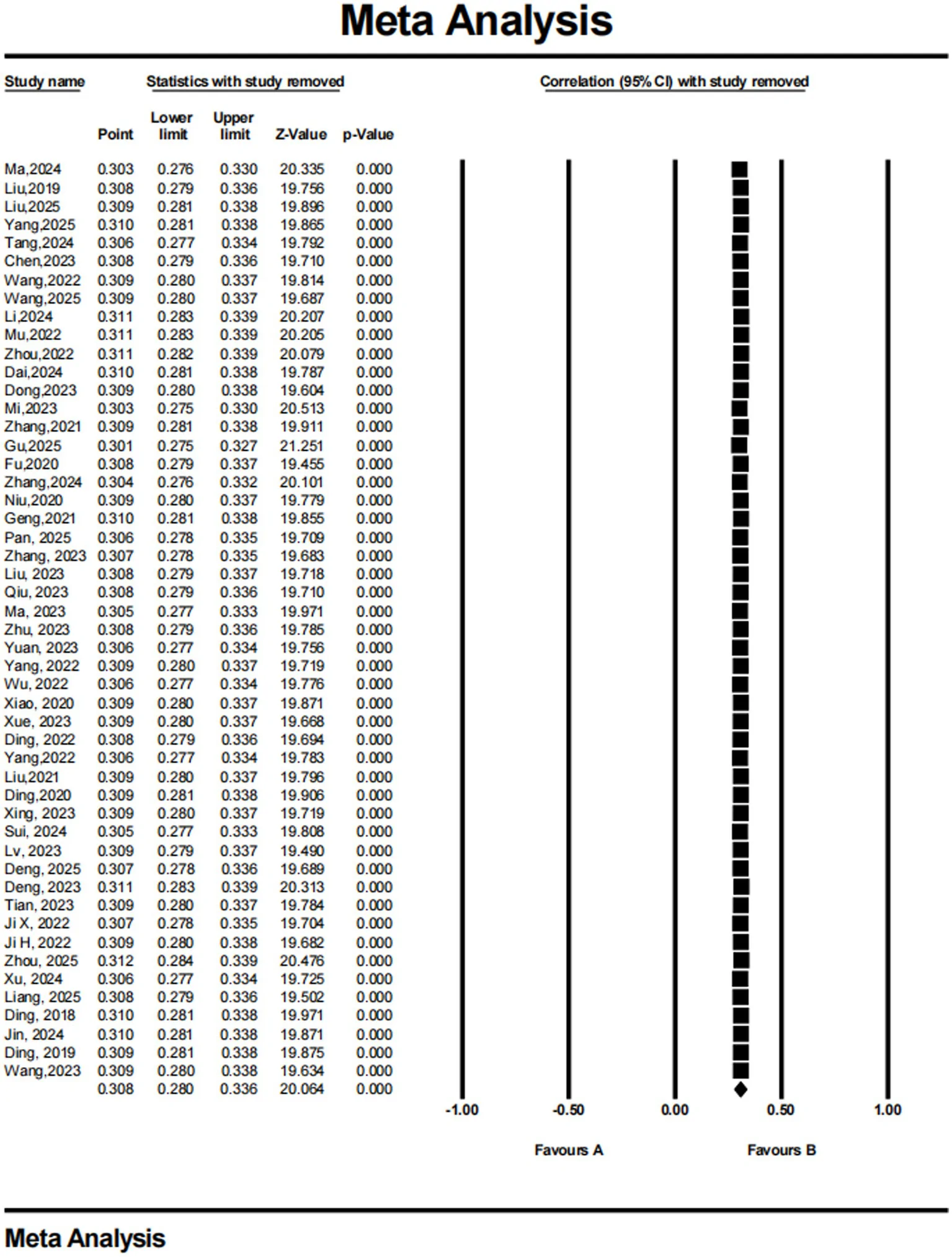
Forest plot for robustness test of male children and adolescents proportion.

Meta-regression results indicated that no significant moderating effect of the proportion of males among children and adolescents was detected for the association between parental phubbing and pathological digital use among children and adolescents. With the proportion of males among children and adolescents taken as a continuous moderator, meta-regression was performed based on 50 independent effect sizes. The regression coefficient for the proportion of males predicting the effect size was non-significant (b = 0.003, SE = 0.003, Z = 1.04, *p* = 0.298, and 95% CI [−0.003, 0.008]), and the overall model test also showed non-significant results (Q = 1.08, df = 1, and *p* = 0.298). Hypothesis 3 was not supported. Leave-one-out sensitivity analysis was performed to test the robustness of the moderating effect of the proportion of males among children and adolescents. As shown in Figure 5, each independent study was sequentially excluded and the overall pooled correlation coefficient was recalculated. Point estimates of pooled effect sizes fluctuated only within a narrow range of 0.301–0.312, and none of the corresponding 95% confidence intervals contained zero. *p*-values for all recalculated overall effects remained below 0.001. Pooled effect sizes exhibited limited fluctuation after sequential exclusion of individual studies, with no material shift observed in the overall association. Such patterns offered preliminary indications that the finding of non-significant moderating effect of the proportion of males may be relatively insensitive to the influence of any single study.

**Figure 6:**
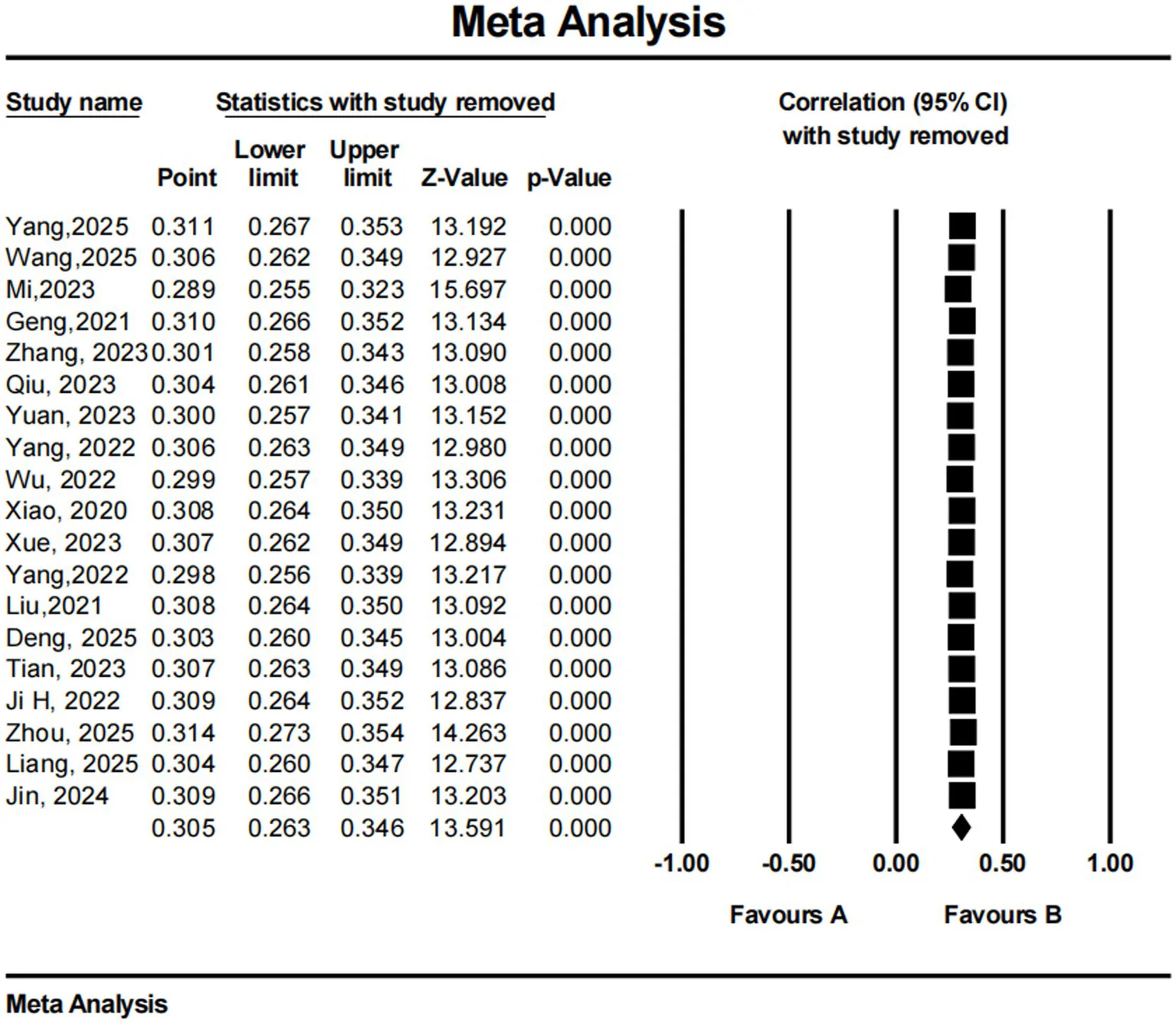
Forest plot of effect sizes for only-child proportion and adolescent proportion.

Meta-regression analysis was conducted based on 19 independent effect sizes due to missing only-child proportion data in some primary studies. The results indicated that no significant moderating effect of only-child proportion on the association between parental phubbing and pathological digital use among children and adolescents was detected (b = 0.0002, SE = 0.0012, Z = 0.13, *p* = 0.894, and 95% CI [−0.002, 0.002]; overall model test: Q = 0.02, df = 1, and *p* = 0.894). Hypothesis 4 was not supported. Leave-one-out sensitivity analysis was conducted to verify the robustness of the moderating effect of only-child proportion. As shown in Figure 6, each eligible study was sequentially excluded, and the overall pooled correlation coefficient was recalculated. Point estimates of pooled effect sizes fluctuated only within a narrow range of 0.289–0.314, and none of the corresponding 95% confidence intervals crossed zero. *p*-values for all recalculated overall effects remained below 0.001. Sequential removal of eligible studies yielded slight changes in pooled effect sizes, and the overall association remained stable. These trends tentatively suggested that the non-significant moderating result for only-child proportion was barely affected by individual studies.

The type of parental phubbing measurement tools significantly moderated the association between parental phubbing and pathological digital use among children and adolescents. After removing subgroups with fewer than three effect sizes, a total of 41 independent effect sizes were included in the subgroup analysis. The between-group heterogeneity test was significant (Q = 6.540, df = 2, and *p* = 0.038), indicating that the strength of the association between parental phubbing and pathological digital use among children and adolescents varied significantly across different measurement tools. Hypothesis 5 was supported.

The type of adolescent pathological digital use measurement tools significantly moderated the association between parental phubbing and pathological digital use among children and adolescents. The initial dataset contained 50 independent effect sizes; effect sizes belonging to subgroups with fewer than three effect sizes were excluded, and a total of 26 independent effect sizes were retained for the subgroup analysis. The between-group heterogeneity test was significant (Q = 11.892, df = 4, and *p* = 0.018). Hypothesis 6 was supported. In addition to measurement tools, further subgroup analysis was conducted with subtypes of pathological digital use (smartphone addiction, Internet addiction, gaming addiction, and short-form video addiction) as the moderator. The mixed-effects model revealed a significant between-group difference (Q = 17.626, df = 3, and *p* = 0.001), demonstrating that the strength of the association between parental phubbing and pathological digital use among children and adolescents differed markedly across various subtypes. Specifically, the smartphone addiction subgroup yielded the largest effect size (*r* = 0.331, and 95% CI [0.297, 0.364]), followed by the short-form video addiction subgroup (*r* = 0.246, 95% CI [0.095, 0.386]) and the Internet addiction subgroup (*r* = 0.244, 95% CI [0.188, 0.299]), while the gaming addiction subgroup produced the smallest effect size (*r* = 0.205, 95% CI [0.149, 0.260]).

An exploratory random-effects meta-regression was performed to examine residential setting (urban versus rural) as a potential moderator. Among all included primary studies, complete information on participants’ residential distribution was available in only nine independent effect sizes, forming the analytical sample for this moderation analysis. The regression coefficient for rural subgroups was −0.001 (Z = −1.16 and *p* = 0.248). The omnibus test of the moderator yielded Q = 1.34 (df = 1 and *p* = 0.2478), revealing that no significant moderating effect of residential setting on the association between parental phubbing and pathological digital use among children and adolescents was detected.

## 4 Discussion

### 4.1 The association between parental phubbing and pathological digital use among children and adolescents

Pooled analyses demonstrated a significant moderate positive association between parental phubbing and pathological digital use. This finding supports Hypothesis 1 and is consistent with the conclusions of most studies (Liu et al., 2025).

From the perspective of parental acceptance-rejection theory, parental phubbing can be viewed as a form of negative parenting, which may reflect potential signs of emotional neglect to some degree. Long-term exposure to such parenting contexts is associated with adverse outcomes for children and adolescents’ emotional development (Zhang Y. et al., 2024; Zhang H. et al., 2024). When emotional deficiency emerges in real-life situations, individuals tend to seek other channels to fill inner emotional gaps. In the digital era, children and adolescents are inclined to satisfy their unmet psychological needs by using digital devices, and this psychological process could be regarded as one potential explanatory pathway through the lens of compensatory Internet use theory (Wei et al., 2022).

### 4.2 Moderating effect analysis

The conclusions drawn from this meta-analysis do not negate the findings obtained in some independent empirical studies. Rather, this meta-analysis only focused on the bivariate association between parental phubbing and pathological digital use among children and adolescents, and potential moderators of pathological digital use may operate within this linkage. Prior to formal data coding, several moderators were pre-specified to test their moderating effects: measurement tools of parental phubbing, measurement tools of adolescent pathological digital use, the proportion of male adolescent participants, the proportion of only-child participants, and age groups of children and adolescents. Given the high heterogeneity detected among pooled effect sizes, an exploratory moderation analysis was additionally performed incorporating residential setting (urban vs. rural). The results showed that the following:

No significant moderating effect of age groups on the association between parental phubbing and pathological digital use among children and adolescents was observed, and Hypothesis 2 was not supported. As a tentative theoretical interpretation of this result, from the perspective of parental acceptance-rejection theory, emotional neglect associated with parental phubbing may bring similar emotional experiences to children and adolescents across different age stages (Rohner and Lansford, 2017). Individuals’ inner needs to alleviate negative emotions and make up for emotional deficiency through online channels may stay relatively comparable across distinct age brackets. The above interpretations remain merely theoretical conjectures requiring further empirical testing.

The proportion of males among children and adolescents did not significantly moderate the association between parental phubbing and pathological digital use among children and adolescents. Some tentative conjectures may account for this non-significant result. In the current digital era, digital devices have been deeply integrated into children and adolescents’ daily lives and learning activities; tasks such as homework submission, activity notifications, and knowledge acquisition all largely rely on digital devices (Alam et al., 2025). Boys and girls generally enjoy comparable access to digital devices, a pattern that may offer a tentative theoretical account for the non-significant moderating role of male proportion. The meta-analysis results of the present study further align with prior evidence that the likelihood of children and adolescents exhibiting pathological digital use shows no significant differences across different proportions of males.

This study found no significant moderating effect of the proportion of only children on the association between parental phubbing and pathological digital use among children and adolescents, a finding that fails to support Hypothesis 4. This result is consistent with the findings of several empirical studies, which indicate no significant difference in the association between parental phubbing and pathological digital use among children and adolescents across groups with different proportions of only children (Wang et al., 2024).

Measurement instruments for parental phubbing significantly moderated the association between parental phubbing and pathological digital use among children and adolescents, a finding consistent with Hypothesis 5. Among the included scales, the revised scale developed by Ding et al. (2020) was associated with a stronger association between the two variables. The original Partner Phubbing Scale by Roberts and David (2016) also showed a significant moderating effect but produced a comparatively smaller correlation coefficient. When completing Roberts’ original scale, adolescents need to extrapolate the interaction context of romantic partners to daily parent–child interactions. As the original scale covers a broader range of scenarios, it may contain behavioral items irrelevant to parent–child exchanges, making its measurement precision lower than that of scales specifically designed for parent–child contexts. Revised by Ding et al. (2020), the scale may achieve higher measurement precision, as its items are adapted to parent–child contexts grounded in the realities of local Chinese families and irrelevant confounding contents are eliminated. This implies that future researchers should adopt localized, parent–child-specific scales for parental phubbing measurement to improve measurement accuracy.

Pathological digital use measurement tools significantly moderated the association between parental phubbing and pathological digital use among children and adolescents, verifying Hypothesis 6. Samples were categorized into five subgroups according to distinct pathological digital use scales. Such discrepancies in effect size magnitude may be attributed to two sources. On the one hand, the degree of alignment between measurement dimensions and theoretical constructs varies across scales. On the other hand, inherent confounding exists between measurement scales and subtypes of pathological digital use. Building on this, further subgroup analysis was performed according to subtypes of pathological digital use. The results showed that the association between parental phubbing and pathological digital use differed significantly across subtypes. This finding is consistent with the study conducted by Baggio et al. The research notes significant discrepancies in effect sizes across smartphone addiction, Internet addiction, gaming addiction, and short-form video addiction and holds that technology-mediated pathological digital use behaviors are relatively independent constructs rather than a fully homogeneous single entity (Baggio et al., 2018). This finding suggests that conceptual differences across behavioral subtypes contribute to the high between-study heterogeneity. However, these distinct subtypes of pathological digital use share common core pathological mechanisms, which provides a theoretical basis for treating them as an integrated construct.

Residential setting (urban vs. rural) showed no significant moderating effect on the association between parental phubbing and pathological digital use among children and adolescents. A tentative interpretation could be drawn based on the current findings and the context of digital development in China. Specifically, the Internet coverage rate of primary and secondary schools has reached 98.4% (Zhu et al., 2022). Rural children and adolescents may utilize smartphones and various online platforms for online learning and entertainment, with a level of digital resource accessibility broadly comparable to that of urban children and adolescents.

Although the moderation analysis identified measurement tools as a significant moderator that could partially explain between-study variability, substantial unexplained between-study variability remains. Such unexplained variability may stem from inconsistent measurement scales for pathological digital use and divergent age groups criteria for children and adolescents across primary studies. Therefore, the overall pooled effect size should be interpreted with caution.

### 4.3 Practical implications

The integrated data synthesized from 50 included empirical studies reveal a moderate positive association between parental phubbing and pathological digital use among children and adolescents, and these correlational findings could offer implications for families, schools, and subsequent researchers. First, suggestions for family practice. The pooled effect size demonstrates a positive association between parental phubbing and pathological digital use among children and adolescents. To alleviate the detrimental effects arising from this association, parents could act as role models by setting fixed electronic-device-free periods during parent–child interactions to demonstrate self-regulation of media use. Second, schools could establish collaborative home-school education channels. Educators could share with guardians the consistent positive association between parental phubbing and pathological digital use among children and adolescents documented in existing empirical research and cooperate with families to deliver emotional support for children and adolescents. Meanwhile, schools could integrate content concerning digital media use and parent–child communication into mental health courses, guiding Chinese children and adolescents to objectively view parental phubbing in daily parent–child interactions within the digital era and independently regulate their personal digital engagement. Finally, methodological guidance for future researchers. The results of this meta-analysis indicate that measurement instruments significantly moderate the association between the two variables. When conducting follow-up empirical investigations on parental phubbing, scholars may prioritize scales specifically developed for Chinese parent–child contexts to accurately assess the severity of parental phubbing within family interactions, rather than scales originally designed for romantic partners.

## 5 Research limitations

Previous studies have failed to reach consistent conclusions on the association between parental phubbing and pathological digital use among children and adolescents. This meta-analysis revealed a significant moderate positive association between the two variables; however, the present study still has several limitations.

First, the pooled correlation coefficient between the two variables rests on two untested assumptions: first, that the association between the two variables follows a linear pattern across the full score range of the scales, and second, that the conceptual connotation of the parental phubbing construct remains stable across both low and high scale score ranges. Accordingly, the moderate correlation effect size obtained in this study should be interpreted on the basis of these assumptions. Future studies are recommended to adopt person-centered analytical approaches (e.g., latent profile analysis), non-linear models (e.g., spline regression), and item response theory to examine scale measurement properties, so as to further explore the heterogeneity of the association at different severity levels of parental phubbing.

Second, all eligible studies only reported Pearson’s correlation coefficients without providing t-values, *F*-values, or chi-square statistics for effect size conversion. This may reduce the representativeness of the pooled sample. Meanwhile, correlation coefficients merely reflect the linear covariation between variables. They cannot adjust for confounding variables, identify the causal direction between parental phubbing and pathological digital use among children and adolescents, or capture the mediational structures described by relevant theoretical frameworks, thus limiting the causal explanatory power of the present findings.

Third, this study only analyzed measurement tools for parental phubbing, measurement tools and behavioral subtypes of pathological digital use, sex of children and adolescents, age groups of children and adolescents, residential setting (rural versus urban), and the proportion of only-child participants. Other potential moderating variables, such as maternal educational attainment, teachers’ academic background, and teaching years, remain to be further investigated in subsequent studies.

In addition, in the subgroup analysis of the moderating effect of measurement tools, subgroups with fewer than three effect sizes were excluded to ensure the reliability and stability of research results. Hence, whether less commonly used measurement tools could moderate the association between parental phubbing and pathological digital use among children and adolescents needs further verification in subsequent studies.

Furthermore, the samples included in this study were mainly from China. Given the limited regional coverage and cultural specificity of the sample, the research conclusions should be applied cautiously in regions with different cultural environments and economic development levels. Unique cultural and institutional factors in China may moderate this association. Chinese children and adolescents face heavy exam-oriented academic pressure, and the widespread tiger parenting style may easily trigger parent–child conflicts over screen use. Such contextual differences may limit cross-cultural generalization of the current findings.

Meanwhile, this study only included children and adolescents with typical development and excluded samples with special educational needs and developmental disorders. Therefore, the research conclusions may not be fully generalized to children and adolescents with special needs such as autism and attention deficit hyperactivity disorder.

Additionally, this study exhibited extremely high overall heterogeneity (I^2^ = 92.191%), with a 95% prediction interval of [0.097, 0.519] that was far broader than the corresponding 95% confidence interval. This indicates that the effect size of the association between parental phubbing and pathological digital use among children and adolescents may fluctuate substantially across future replications. Therefore, the generalizability of the present findings must be interpreted cautiously in light of specific research contexts.

Another limitation involves publication bias. Journals tend to publish studies reporting significant adverse effects of parental phubbing on children and adolescents, whereas non-significant findings are underrepresented, which may inflate the pooled effect size. The trim-and-fill method imputed two missing studies. After adjustment, the pooled effect size slightly declined from *r* = 0.308 (95% CI [0.280, 0.336]) to *r* = 0.295 (95% CI [0.263, 0.326]). Statistical significance remained unchanged, demonstrating the robustness of the core findings. Future studies are recommended to adopt PROSPERO pre-registration and registered reports and fully report all outcomes to reduce selective reporting bias.

Regarding sample age stratification, participants included in the analysis were aged 6 to 18 years, spanning both childhood and adolescence. Moderator analyses revealed no significant age differences; however, comprehensive stratified subgroup comparisons across distinct age brackets were not performed. A substantial proportion of primary studies lacked clear, uniform age categorization and were thus excluded from age-based subgroup tests. This exclusion may reduce statistical power and limit the precision of the overall findings. Subsequent meta-analyses could disaggregate child and adolescent samples to examine divergent developmental patterns.

Finally, all participants were general primary and secondary school students without prior screening, and no systematic psychiatric screening was conducted to exclude participants with subclinical anxiety and depressive symptoms. Only studies recruiting clinically diagnosed populations were excluded from the analysis. Considering that the absence of standardized clinical screening may introduce confounding bias into the pooled effect sizes, this constitutes an important limitation of the present meta-analysis.

## 6 Conclusion

This meta-analysis synthesized existing empirical studies and preliminarily confirmed a statistically significant, moderate positive association between parental phubbing and pathological digital use among children and adolescents. Nevertheless, notable between-study heterogeneity was observed, indicating that the true effect size may vary across different research contexts and sample characteristics. The findings provided preliminary evidence supporting the applicability of parental acceptance-rejection theory, compensatory Internet use theory, and other relevant theories in this research domain. Moderation analyses showed that measurement tools for parental phubbing, measurement tools for pathological digital use, and subtypes of pathological digital use exerted significant moderating effects on this association. In contrast, age groups, the proportion of only children, the proportion of male participants, and residential setting (urban vs. rural) showed no significant moderating effects. Since the examined moderators only partially accounted for the between-study heterogeneity, unidentified sources of effect size variation remained. Accordingly, the pooled effect size had limited generalizability and could not be unconditionally extended to all populations and research contexts.

## Data Availability

The original contributions presented in the study are included in the article/Supplementary material, further inquiries can be directed to the corresponding author.
